# The basal bodies of *Chlamydomonas reinhardtii*

**DOI:** 10.1186/s13630-016-0039-z

**Published:** 2016-06-01

**Authors:** Susan K. Dutcher, Eileen T. O’Toole

**Affiliations:** Washington University at St. Louis, St. Louis, USA; University of Colorado at Boulder, Boulder, USA

**Keywords:** Site-specific basal body duplication, Cartwheel, Transition zone, Centrin fibers

## Abstract

The unicellular green alga, *Chlamydomonas reinhardtii*, is a biflagellated cell that can swim or glide. *C. reinhardtii* cells are amenable to genetic, biochemical, proteomic, and microscopic analysis of its basal bodies. The basal bodies contain triplet microtubules and a well-ordered transition zone. Both the mother and daughter basal bodies assemble flagella. Many of the proteins found in other basal body-containing organisms are present in the *Chlamydomonas* genome, and mutants in these genes affect the assembly of basal bodies. Electron microscopic analysis shows that basal body duplication is site-specific and this may be important for the proper duplication and spatial organization of these organelles. *Chlamydomonas* is an excellent model for the study of basal bodies as well as the transition zone.

## Phylogeny and conservation of proteins

The green lineage or Viridiplantae consists of the green algae, which include *Chlamydomonas*, the angiosperms (the land plants), and the gymnosperms (conifers, cycads, ginkgos). They are grouped together because they have chlorophyll a and b and lack phycobiliproteins. The green algae together with the cycads and ginkgos have basal bodies and cilia, while the angiosperms and conifers have lost these organelles. The green algae are often referred to as bikonts because they have two flagella. A unikont (eukaryotic cells with a single flagellum) is thought to be the ancestor of choanoflagellates, fungi, and animals [6]. The uniflagellated green alga *Micromonas* is an example of a unikont [40]. It is possible to identify mutants in three genes in *Chlamydomonas* that assemble only one flagellum [13, 25, 42, 45, 46].

Many of the basal body and flagellar proteins are conserved across a wide range of organisms by sequence comparisons [1, 33]. A small number of proteins identified by mutational analysis in a variety of organisms are needed to assemble the microtubule core of basal bodies; these include PLK4, SAS6, SAS4, BLD10/CEP135, POC1, Centrin, SPD2/CEP192, Asterless/CEP152; CEP70, delta-tubulin, and epsilon-tubulin. *Chlamydomonas* has homologs of all of these based on sequence conservation except PLK4, CEP152, and CEP192. Several lines of evidence suggests that CEP152, CEP192, and PLK4 interact [20, 52] and their concomitant absence in several organisms suggests that other mechanisms exist that allow for control of duplication [4]. For the conserved proteins, mutations or knockdown of SAS6, BLD10/CEP135, Centrin, CEP70, delta-tubulin, and epsilon-tubulin affect basal body duplication and assembly in *Chlamydomonas* as has been observed in other organisms. The proteomic analysis of basal bodies from *Chlamydomonas* [30] identified multiple POC (proteome of centriole) proteins and many have homologs (see below). Because of this conservation in proteins and structure as described below, *Chlamydomonas* remains an important model organism for basal body and flagellar research.

## Basal body structure

**Fig. 1:**
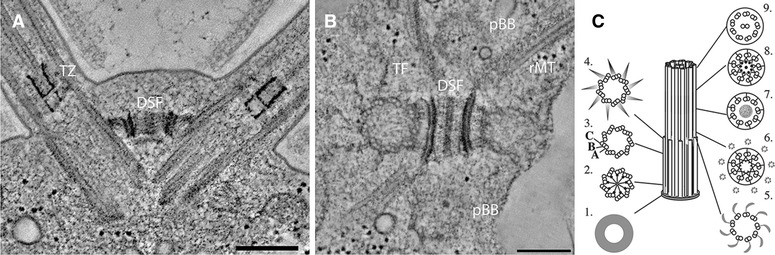
Electron tomography of *Chlamydomonas* basal bodies reveals characteristic 3D organization. **a** Mature basal bodies template the microtubules of the flagella and are held together at the distal end by a distal striated fiber. The transition zone appears as an electron dense H-shaped structure in longitudinal view. TZ denotes the transition zone and DSF denotes the distal striated fiber. **b** Cross-sectional view showing distal striated fiber connecting mature basal bodies, two probasal bodies and four bundles of rootlet microtubules in a cruciate arrangement. *DSF* distal striated fiber, *TF* transition fiber, *pBB* probasal body, *rMT* rootlet microtubules. **c** Diagram showing distinct structural features of *Chlamydomonas* basal bodies (Reprinted with permission from Molecular Biology of the Cell; [42]). *Bar* = 200 nm

*Chlamydomonas* cells in interphase have a pair of mature basal bodies and a pair of probasal bodies [5, 22, 29, 48] (Fig. 1). Both mature and probasal bodies have triplet microtubules. The probasal bodies have an average length of 86 nm and the mature basal bodies have an average length of ~400 nm. Both the mature and probasal bodies have a proximal cartwheel. The proximal end of the mature basal bodies contains a ring of amorphous material that is found below the cartwheel [42]. The cartwheel spokes require BLD12/SAS-6 [39] and the spoke tips require BLD10/CEP135 [37]. Spoke tips can still assemble in the absence of BLD12/SAS-6.

Cryo-electron tomography and 3D subtomogram averaging of isolated basal bodies at 33 Å resolution reveal additional densities that represent non-tubulin proteins attached to the triplet microtubules, including a large inner circular structure in the basal body lumen [34]. The basal bodies have also been examined by serial thin-section electron microscopy in an elegant study [22]. This study found evidence of rotational symmetry via a structure called the acorn, which is found at the distal end of the triplet microtubules as they transition to the doublet microtubules of the transition zone. It is a filament of about 10 nm that is present on the A-tubules of doublets 1, 7, 8, and 9.

The cartwheel is very dynamic during mitosis. The length of the cartwheel undergoes significant changes during the cell cycle [3]. The cartwheel length in new probasal bodies that assemble during metaphase is ~107 nm and increases to ~163 nm in telophase. The elongated cartwheel may act to stabilize this forming probasal body [41]. In addition, the elongation of the cartwheel is concurrent with the elongation of triplet MTs of the daughter basal body prior to mitosis. Similar to the triplet MTs, the cartwheel in the developing daughter basal body during elongation is also significantly longer than in interphase (137 + 23 vs. 42 + 5 nm, respectively). This additional length could become the excised SAS-6 structure that templates new centrioles as has been observed in mammalian cells [21].

**Fig. 2:**
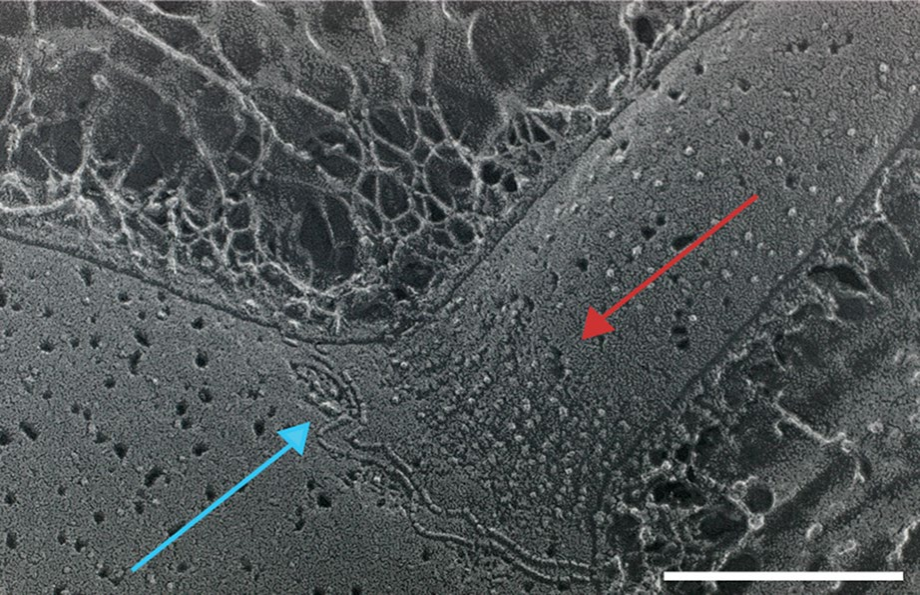
Quick-freeze deep-etch electron microscopy showing the ciliary necklace composed of many intramembranous particles (*red arrow*) and the ciliary bracelet (*blue arrow*). *Bar* = 200 nm

The transition zone, which is ~135 nm long, contains an elaborate stellate pattern in the interior of the microtubule barrel [22, 43, 48] (Fig. 1). These stellate patterns are observed as an osmophilic H-structure in longitudinal TEM sections (Fig. 1, TZ). In the transition zone, the Y-linkers are positioned between the microtubule core and the ciliary membrane. This is the region where CEP290 and NPHP4 localize [2, 7]. In the ciliary membrane there are two sets of intramembranous particles. These are the ciliary necklace and the ciliary bracelet [57] (Fig. 2). The composition of the ciliary necklace and bracelet is not known. The ciliary bracelet has not been observed in other organisms.

The analysis of the *uni3* mutant was instrumental in the identification of new tubulin isoforms [13]. The *Chlamydomonas* genome encodes delta, epsilon, and zeta tubulin isoforms. The loss of δ-tubulin results in the loss of triplet microtubules proximally [13, 42], and the loss of ε-tubulin results in the loss of doublet and triplet microtubules [15, 18, 47]. Antibodies to ε-tubulin showed a ring around the basal bodies as well as two projections coming off of each basal body that overlap the rootlet microtubule. Mutants of ζ-tubulin have not been identified [11].

## Other structures and fibers

**Fig. 3:**
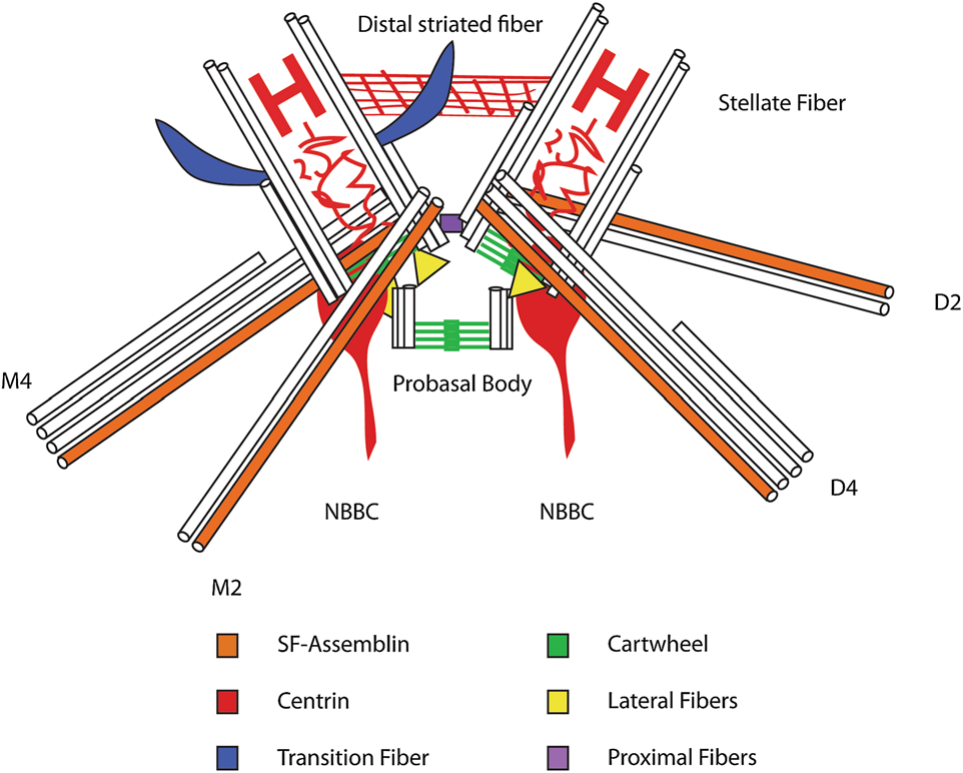
Fibers attached to the basal bodies. The mature basal bodies and the probasal bodies are shown as white microtubules with the cartwheel at the proximal region shown in *green*. The two mature basal bodies are found at an oblique angle to one another and are about 400 nm in length. They are connected at their distal end by the distal striated fiber (*red*) and connected at the proximal end by the proximal striated fiber (*purple*). Lateral fibers (*yellow*) connect the mature basal body to its daughter probasal body via the VFL3 protein. Centrin (in *red*) is shown in the lumen of the basal body, in the stellate fiber, and in the nucleo-basal body connector (NBBC). The four-rootlet microtubules are shown with the four-membered (M4 and D4) and the two-membered (M2 and D2). One of the rootlet microtubules is associated with a striated fiber that contains SF-assemblin (*orange*). The transition fibers (*blue*) are shown on one of the basal bodies but each basal body has nine transition fibers. A microtubule is about 25 nm as an internal *scale bar*

The basal bodies have a variety of associated structures as shown in Fig. 3.

### Transition fibers

These fibers initiate at the beginning of the transition zone when the triplet microtubules become doublet microtubules. The basal body must be anchored to the plasma membrane, and this occurs via transition fibers [5, 12, 48]. The transition fibers are striated and end in knobs at the plasma membrane [43] and are likely to play roles similar to the distal and subdistal appendages of mammalian cells. The transition fibers may also serve as a docking site for intraflagellar transport (IFT) proteins [8]. Of the appendage proteins found in mammals, the *Chlamydomonas* genome has only CEP164 and CC2D2A.

### Rootlet microtubules

The mother and daughter basal bodies are each associated with two microtubules bundles called the rootlet microtubules. Each basal body has a four-membered microtubule and a two-membered microtubule rootlet. The rootlet microtubules are stable and contain acetylated α-tubulin. The rootlets are attached to the basal bodies at specific triplet microtubules and remain associated with the basal bodies during both interphase and mitosis. These microtubules form a cruciate pattern (Fig. 1b).

The rootlet microtubules attached to the basal bodies have at least two functions. The four-membered rootlets mark the cleavage furrow [24]. In mutants with defective basal bodies, the rootlets lose their spatial organization and the cleavage furrow is misplaced relative to the spindle [18, 39]. The four-membered rootlet on the daughter basal body (D4) is also involved in placement of the photosensory eyespot at the end of the mitotic cell cycle. MLT1 localizes to D4 and centrin excludes it from the two-membered daughter rootlet (D2) [38]. Centrin is involved in excluding MLT1 from D2.

### SF-assemblin

Striated fibers overlay the two-membered rootlet microtubules and contain the coiled-coil protein, SF-assemblin [32]. SF-assemblin staining is reduced to two dot-like structures at each spindle pole in metaphase, and at telophase new fibers assemble.

### Proximal fibers

The composition of these fibers is unknown but they also appear as striated fibers. Two proximal fibers attach the mother and daughter basal body at their proximal ends.

### Centrin fibers

This is a calcium-binding protein with EF-hand domains and it is found in several basal body-associated structures. This protein is widely conserved in eukaryotes [4]. Centrin is also found in two patches on the anterior ends of the two-membered microtubule rootlets on the probasal bodies, in the lumen of the basal body, and in the stellate fiber of the transition zone [22]. In addition, centrin is found in the distal striated fibers, and the nucleo-basal body connector.

### Distal striated fibers

The mother and daughter basal bodies are connected by a large fiber called the distal striated fiber (DSF), which contain centrin (Fig. 1). The DSF disassembles in mitotic prophase so that the mother and daughter basal bodies can move apart to the opposite sides of the nuclear envelope [31, 41]. The DSF requires triplet microtubules to assemble or attach. Mutations that affect the assembly of triplet microtubules have defects in the assembly/attachment of the DSF [42].

### Nucleo-basal body connector

The two NBBCs extend from both basal bodies and connect basal bodies with the nucleus [58]. Centrin missense mutations result in misorientation and missegregation of the basal bodies [36, 58] that result in a variable number of flagella. It is not clear if this is due to the loss of the DSF, the NBBC, or both.

## Basal body duplication

In interphase of the mitotic cell cycle, the basal bodies are attached to the plasma membrane and both the mother and the daughter basal bodies assemble flagella. At the beginning of mitosis, the transition zone is severed from the basal body at the proximal end of the transition zone ([41, 44]. The probasal bodies are elongated and new probasal bodies are initiated in association with the two older basal bodies in prophase/metaphase. The new probasal body appears next to triplet microtubules 7 and 8 of the mature basal bodies. This suggests that there is a unique site that is licensed for duplication in a wild-type cell. The new probasal bodies first assemble singlet microtubules from the cartwheel structure. During telophase/cytokinesis when the doublet and triplet microtubules assemble, the microtubules assemble from the distal to the proximal end of the new probasal bodies [41]. PLK4, which is not present in the *Chlamydomonas* genome, forms a single spot that precedes the duplication of centrioles in mammalian cells [51]. It has been suggested that this localization provides a site for duplication prior to the recruitment of SAS6.

The basal bodies are found near the poles of the spindle, but they do not appear to act as microtubule organizing centers for astral or spindle microtubules. The older basal bodies remain associated with the plasma membrane [22, 41]. At telophase, the *Chlamydomonas* cell has four mature basal bodies and four probasal bodies. In centrin mutants (*vfl2*), segregation of the basal bodies is perturbed as discussed above. Cells with no basal bodies and with too many basal bodies have been observed in the centrin mutants [36] or epsilon-tubulin mutant (*bld2*) [18]. In the *bld2* mutant, the spindles appear normal. Basal bodies can be regenerated de novo in the cells lacking basal bodies [36].

There are conflicting results about the presence of basal bodies during meiosis [5, 54]. Triemer and Brown observed the presence of a pair of basal bodies near the nucleus and plasma membrane. Several basal bodies mutants [*bld2* (ε-tubulin) and *bld10* (CEP135)] have recessive meiotic defects that suggest that basal bodies are needed for the successful completion of meiosis, but it is not known what role these basal bodies play.

## Identification of basal body components

Proteomics was performed on isolated basal bodies [30]. In these preparations, chloroplast and mitochondrial proteins accounted for 55/195 proteins identified; some of the unidentified proteins may be from these organelles. Forty-five of the proteins were validated by examining localization to the centrioles in mammalian cells. mRNA levels were increased for 27 proteins and these were named BUG1-BUG27. Several of these are known to have roles in the flagella [27, 28, 50, 59]. Eighteen proteins (POC1-POC18) were postulated to be part of the basal body complex. Two-dimensional gel electrophoresis of basal bodies [10] reveals over 200 proteins in isolated preparations; additional proteomic analysis may be useful to identify additional proteins.

In mitosis, the transition zones are discarded from the basal bodies by severing that occurs at the proximal end of the transition zone [44]. Proteomics of these transition zone remnants have many of the proteins known to be in the transition zone from other organisms. These include CEP290, NPHP1, NPHP4, MKS1, MKS3/TMEM67, MKS6, Tectonic, AHI1, as well as several BBS proteins. The novel proteins that were identified by proteomics are ESCRT proteins. These include VPS4, VPS23, VPS28, VPS37, VPS60, and the exosome maturation protein ALIX/PCDP6IP [9]. Both subunits of katanin were also found [16, 17, 19]. An aminophospholipid transporter is present as well as two proteins with lipoxygenase homology domains (POC2 and MOT51). The cell cycle proteins, CDKA1 and CYCA1, are also present. Mutants of CDKA1 are required to initiate DNA replication in *Chlamydomonas* [55]. 33 novel proteins were also identified.

Examination of mRNA levels using synchronized cells shows that most of the known basal body genes, *POC* genes, and 4 *BUG* genes (BUG18, 23, 24, 27) show a peak increase in their mRNA levels just before cells enter into mitosis [60]. The levels of ε-tubulin, γ-tubulin, *BUG25*, a coiled-coil protein, *POC7* (*UNC*-*119*), and *MKS1* mRNA increase earlier when many of the genes involved in DNA replication increase. δ-tubulin levels remain high until the cells reenter interphase with the introduction of light. Most of the BUG genes show patterns that are more similar to the pattern observed for flagellar genes. The cluster that is enriched in known basal body and POC genes contain over 1000 transcripts. These may encode additional proteins needed for basal body and transition zone assembly. As might be expected, the ESCRT proteins found in the transition zone are not in the cluster enriched in basal body genes as they are likely to have multiple functions.

## Notable basal body findings

The *bld2* mutant was the first mutant identified that fails to assemble basal bodies and this result suggested that basal bodies were not essential in *Chlamydomonas* [14, 23]. The *uni3* mutant, which is a deletion of δ-tubulin, provided the first evidence that additional tubulin isoforms are needed for basal body assembly and maintenance [13, 42], and this was supported by the identification that the *BLD2* encodes ε-tubulin. The *Chlamydomonas BLD12/SAS6* protein was used in the crystallization of SAS6 ([56]). Evidence for *de novo* assembly of basal bodies came from using *vfl* mutants [36]. The calcium-binding protein, centrin, was identified in *Chlamydomonas* [26, 49]. A missense mutation in one of the EF-hand domains of centrin results in loss of several of the basal body-associated fibers [53], while knockdown of the centrin gene resulted in a failure to duplicate basal bodies [31]. Comparative genomics using *Chlamydomonas* and *Drosophila* was a key to identifying many basal body and flagellar proteins. This lead to the realization that the proteins mutated in Bardet–Biedl Syndrome (BBS) affected flagellar function [1, 33]. Isolation of basal bodies lead to the identification of *POC* genes [30]. Isolation of transition zone remnants allows for a further proteomic characterization of this important structural region and the finding that multiple ESCRT proteins at this key structure [9]. The site-specific duplication of new basal bodies on specific microtubules was demonstrated in *Chlamydomonas* [41].

## Strengths and future of basal body research in *Chlamydomonas*

The strength of *Chlamydomonas* has come from the ability to use multiple approaches. These include genetics, microscopy, and biochemistry. *Chlamydomonas* strains are haploid, which makes forward genetics straightforward. In addition, diploid strains can be selected and used for complementation tests. The power of whole genome sequencing has made it easier to use forward genetics to identify new mutations that affect the structure and function of basal bodies and transition zone and then to identify the gene of interest by whole genome sequencing [35]. Most of the known basal body mutants have defects in flagellar assembly; so rapid screens for cells that fail to swim properly can be performed. Basal body mutants that have defects in the triplet microtubules confer supersensitivity to Taxol, so secondary screens can be performed to identify structural mutants [19]. Screens for transition zone mutants are also possible using similar strategies. These mutants can be studied by electron tomography, quick-freeze deep-etch electron microscopy and by proteomics to further understand the assembly pathways for basal bodies and organelles. Since basal bodies are essential to flagellar assembly and function, these studies must include the analysis of the flagellar composition and function as well.

## Abbreviations

Å: Angstrom, DSF: Distal striated fiber, NBBC: Nucleo-basal body connector, IFT: Intraflagellar transport, D2: Rootlet microtubule associated with daughter basal body with two microtubules, D4: Rootlet microtubule associated with daughter basal body with four microtubules, M2: Rootlet microtubule associated with mother basal body with two microtubules, M4: Rootlet microtubule associated with mother basal body with four microtubules.

### Genetic Acronyms

BBS: Bardet–Biedl Syndrome, BUG: Basal body upregulated gene, BLD: Bald, CC2D2A: Coiled-Coil And C2 Domain Containing 2A, CEP: Centriole proteome, MLT: Multieye, MOT: Present in motile ciliated organisms, MKS: Meckel’s Syndrome, NPHP: Nephronophthsis, POC: proteome of centrioles, SAS: Spindle assembly abnormal, TMEM: Transmembrane protein, UNI: Uniflagellate, VFL: Variable flagellar number, VPS: Vacuolar Protein Sorting-Associated Protein.

## References

[1] Avidor-Reiss T, Maer AM, Koundakjian E, Polyanovsky A, Keil T, Subramaniam S, Zuker CS (2004). Decoding cilia function: defining specialized genes required for compartmentalized cilia biogenesis. Cell.

[2] Awata J, Takada S, Standley C, Lechtreck KF, Bellve KD, Pazour GJ, Fogarty KE, Witman GB (2014). NPHP4 controls ciliary trafficking of membrane proteins and large soluble proteins at the transition zone. J Cell Sci.

[3] Beech PL, Heimann K, Melkonian M (1991). Development of the flagellar apparatus during the cell cycle of unicellular alga Protoplasma..

[4] Carvalho-Santos Z, Azimzadeh J, Pereira-Leal JB, Bettencourt-Dias M (2011). Evolution: tracing the origins of centrioles, cilia, and flagella. J Cell Biol.

[5] Cavalier-Smith T (1974). Basal body and flagellar development during the vegetative cell cycle and the sexual cycle of *Chlamydomonas reinhardii*. J Cell Sci.

[6] Cavalier-Smith T (2009). Megaphylogeny, cell body plans, adaptive zones: causes and timing of eukaryote basal radiations. J Eukaryot Microbiol.

[7] Craige B, Tsao CC, Diener DR, Hou Y, Lechtreck KF, Rosenbaum JL, Witman GB (2010). CEP290 tethers flagellar transition zone microtubules to the membrane and regulates flagellar protein content. J Cell Biol.

[8] Deane JA, Cole DG, Seeley ES, Diener DR, Rosenbaum JL (2001). Localization of intraflagellar transport protein IFT52 identifies basal body transitional fibers as the docking site for IFT particles. Curr Biol.

[9] Diener DR, Lupetti P, Rosenbaum JL (2015). Proteomic analysis of isolated ciliary transition zones reveals the presence of ESCRT proteins. Curr Biol.

[10] Dutcher SK (1995). Purification of basal bodies and basal body complexes from *Chlamydomonas reinhardtii*. Methods Cell Biol.

[11] Dutcher SK (2001). The tubulin fraternity: alpha to eta. Curr Opin Cell Biol.

[12] Dutcher SK, Witman GB (2009). Basal bodies and associated structures. The Chlamydomonas sourcebook.

[13] Dutcher S, Trabuco E (1998). The *UNI3* gene is required for assembly of basal bodies in Chlamydomonas and encodes d-tubulin, a new member of the tubulin superfamily. Mol Biol Cell.

[14] Dutcher S, Morrissette N, Preble A, Rackley C, Stanga J (2002). e-tubulin is an essential component of the centriole. Mol Biol Cell.

[15] Dutcher SK, Morrissette NS, Preble AM, Rackley C, Stanga J (2002). Epsilon-tubulin is an essential component of the centriole. Mol Biol Cell.

[16] Dymek EE, Smith EF (2012). PF19 encodes the p60 catalytic subunit of katanin and is required for assembly of the flagellar central apparatus in Chlamydomonas. J Cell Sci.

[17] Dymek EE, Lefebvre PA, Smith EF (2004). PF15p is the chlamydomonas homologue of the Katanin p80 subunit and is required for assembly of flagellar central microtubules. Eukaryot Cell.

[18] Ehler L, Jai H, Dutcher S (1995). Loss of spatial control of the mitotic spindle apparatus in a *Chlamydomonas reinhardtii* mutant strain lacking basal bodies. Genetics.

[19] Esparza JM, O’Toole E, Li L, Giddings TH, Kozak B, Albee AJ, Dutcher SK (2013). Katanin localization requires triplet microtubules in *Chlamydomonas reinhardtii*. PLoS ONE.

[20] Firat-Karalar EN, Stearns T (2015). Probing mammalian centrosome structure using BioID proximity-dependent biotinylation. Methods Cell Biol.

[21] Fong CS, Kim M, Yang TT, Liao JC, Tsou MF (2014). SAS-6 assembly templated by the lumen of cartwheel-less centrioles precedes centriole duplication. Dev Cell.

[22] Geimer S, Melkonian M (2004). The ultrastructure of the *Chlamydomonas reinhardtii* basal apparatus: identification of an early marker of radial asymmetry inherent in the basal body. J Cell Sci.

[23] Goodenough UW, St. Clair HS (1975). BALD-2: a mutation affecting the formation of doublet and triplet sets of microtubules in *Chlamydomonas reinhardtii*. J Cell Biol.

[24] Holmes J, Dutcher S (1989). Cellular asymmetry in *Chlamydomonas reinhardtii*. J Cell Sci.

[25] Huang B, Ramanis Z, Dutcher SK, Luck DJ (1982). Uniflagellar mutants of Chlamydomonas: evidence for the role of basal bodies in transmission of positional information. Cell.

[26] Huang B, Mengersen A, Lee VD (1988). Molecular cloning of cDNA for caltractin, a basal body-associated Ca2 + -binding protein: homology in its protein sequence with calmodulin and the yeast CDC31 gene product. J Cell Biol.

[27] Ikeda T (2008). Parkin-co-regulated gene (PACRG) product interacts with tubulin and microtubules. FEBS Lett.

[28] Ikeda K, Ikeda T, Morikawa K, Kamiya R (2007). Axonemal localization of Chlamydomonas PACRG, a homologue of the human Parkin-coregulated gene product. Cell Motil Cytoskelet.

[29] Johnson UG, Porter KR (1968). Fine structure of cell division in *Chlamydomonas reinhardi*. Basal bodies and microtubules. J Cell Biol.

[30] Keller LC, Romijn EP, Zamora I, Yates JR, Marshall WF (2005). Proteomic analysis of isolated chlamydomonas centrioles reveals orthologs of ciliary-disease genes. Curr Biol.

[31] Koblenz B, Schoppmeier J, Grunow A, Lechtreck KF (2003). Centrin deficiency in Chlamydomonas causes defects in basal body replication, segregation and maturation. J Cell Sci.

[32] Lechtreck KF, Silflow CD (1997). SF-assemblin in Chlamydomonas: sequence conservation and localization during the cell cycle. Cell Motil Cytoskelet.

[33] Li JB, Gerdes JM, Haycraft CJ, Fan Y, Teslovich TM, May-Simera H, Li H, Blacque OE, Li L, Leitch CC, Lewis RA, Green JS, Parfrey PS, Leroux MR, Davidson WS, Beales PL, Guay-Woodford LM, Yoder BK, Stormo GD, Katsanis N, Dutcher SK (2004). Comparative genomics identifies a flagellar and basal body proteome that includes the BBS5 human disease gene. Cell.

[34] Li S, Fernandez JJ, Marshall WF, Agard DA (2012). Three-dimensional structure of basal body triplet revealed by electron cryo-tomography. EMBO J.

[35] Lin H, Miller ML, Granas DM, Dutcher SK (2013). Whole genome sequencing identifies a deletion in protein phosphatase 2A that affects its stability and localization in *Chlamydomonas reinhardtii*. PLoS Genet.

[36] Marshall WF, Vucica Y, Rosenbaum JL (2001). Kinetics and regulation of de novo centriole assembly. Implications for the mechanism of centriole duplication. Curr Biol.

[37] Matsuura K, Lefebvre PA, Kamiya R, Hirono M (2004). Bld10p, a novel protein essential for basal body assembly in Chlamydomonas: localization to the cartwheel, the first ninefold symmetrical structure appearing during assembly. J Cell Biol.

[38] Mittelmeier TM, Thompson MD, Lamb MR, Lin H, Dieckmann CL (2015). MLT1 links cytoskeletal asymmetry to organelle placement in chlamydomonas. Cytoskeleton..

[39] Nakazawa Y, Hiraki M, Kamiya R, Hirono M (2007). SAS-6 is a cartwheel protein that establishes the 9-fold symmetry of the centriole. Curr Biol.

[40] Omoto CK, Witman GB (1981). Functionally significant central-pair rotation in a primitive eukaryotic flagellum. Nature.

[41] O’Toole E, Dutcher SK (2014). Site-specific basal body duplication in Chlamydomonas cytoskeleton. Cytoskeleton.

[42] O’Toole E, Giddings T, McIntosh J, Dutcher S (2003). Three-dimensional organization of basal bodies from wild-type and d-tubulin deletion strains of *Chlamydomonas reinhardtii*. Mol Biol Cell.

[43] O’Toole ET, Giddings TH, Dutcher SK (2007). Understanding microtubule organizing centers by comparing mutant and wild-type structures with electron tomography. Methods Cell Biol.

[44] Parker JD, Hilton LK, Diener DR, Rasi MQ, Mahjoub MR, Rosenbaum JL, Quarmby LM (2010). Centrioles are freed from cilia by severing prior to mitosis. Cytoskeleton.

[45] Piasecki BP, Silflow CD (2009). The UNI1 and UNI2 genes function in the transition of triplet to doublet microtubules between the centriole and cilium in Chlamydomonas. Mol Biol Cell.

[46] Piasecki BP, LaVoie M, Tam LW, Lefebvre PA, Silflow CD (2008). The Uni2 phosphoprotein is a cell cycle regulated component of the basal body maturation pathway in *Chlamydomonas reinhardtii*. Mol Biol Cell.

[47] Preble AM, Giddings TH, Dutcher SK (2001). Extragenic bypass suppressors of mutations in the essential gene BLD2 promote assembly of basal bodies with abnormal microtubules in *Chlamydomonas reinhardtii*. Genetics.

[48] Ringo DL (1967). Flagellar motion and fine structure of the flagellar apparatus in Chlamydomonas. J Cell Biol.

[49] Salisbury JL, Baron AT, Sanders MA (1988). The centrin-based cytoskeleton of *Chlamydomonas reinhardtii*: distribution in interphase and mitotic cells. J Cell Biol.

[50] Silflow CD, Sun X, Haas NA, Foley JW, Lefebvre PA (2011). The Hsp70 and Hsp40 chaperones influence microtubule stability in Chlamydomonas. Genetics.

[51] Sonnen KF, Schermelleh L, Leonhardt H, Nigg EA (2012). 3D-structured illumination microscopy provides novel insight into architecture of human centrosomes. Biol open.

[52] Sonnen KF, Gabryjonczyk AM, Anselm E, Stierhof YD, Nigg EA (2013). Human Cep192 and Cep152 cooperate in Plk4 recruitment and centriole duplication. J Cell Sci.

[53] Taillon BE, Adler SA, Suhan JP, Jarvik JW (1992). Mutational analysis of centrin: an EF-hand protein associated with three distinct contractile fibers in the basal body apparatus of Chlamydomonas. J Cell Biol.

[54] Triemer R, Brown R (1976). Ultrastructure of meiosis in *Chlamydomonas reinhardtii*. Br Phycol.

[55] Tulin F, Cross FR (2015). Cyclin-Dependent Kinase Regulation of Diurnal Transcription in Chlamydomonas. Plant Cell.

[56] Van Breugel M, Hirono M, Andreeva A, Yanagisawa HA, Yamaguchi S, Nakazawa Y, Morgner N, Petrovich M, Ebong IO, Robinson CV, Johnson CM, Veprintsev D, Zuber B (2011). Structures of SAS-6 suggest its organization in centrioles. Science.

[57] Weiss RL, Goodenough DA, Goodenough UW (1977). Membrane particle arrays associated with the basal body and with contractile vacuole secretion in Chlamydomonas. J Cell Biol.

[58] Wright RL, Salisbury J, Jarvik JW (1985). A nucleus-basal body connector in *Chlamydomonas reinhardtii* that may function in basal body localization or segregation. J Cell Biol.

[59] Yanagisawa HA, Mathis G, Oda T, Hirono M, Richey EA, Ishikawa H, Marshall WF, Kikkawa M, Qin H (2014). FAP20 is an inner junction protein of doublet microtubules essential for both the planar asymmetrical waveform and stability of flagella in Chlamydomonas. Mol Biol Cell.

[60] Zones JM, Blaby IK, Merchant SS, Umen JG (2015). High-resolution profiling of a synchronized diurnal transcriptome from *Chlamydomonas reinhardtii* reveals continuous cell and metabolic differentiation. Plant Cell.

